# Metabolomic-Based Assessment of Earthworm (*Eisenia fetida*) Exposure to Different Petroleum Fractions in Soils

**DOI:** 10.3390/metabo15020097

**Published:** 2025-02-05

**Authors:** Meiyu Liu, Mutian Wang, Xiaowen Fu, Fanyong Song, Fangyuan Zhou, Tianyuan Li, Jianing Wang

**Affiliations:** 1 Shandong Provincial Key Laboratory of Applied Microbiology, Ecology Institute, Qilu University of Technology (Shandong Academy of Sciences), Jinan 250100, China; 10431221199@stu.qlu.edu.cn (M.L.); songfy@qlu.edu.cn (F.S.); fangyuan_zhou@qlu.edu.cn (F.Z.); 707027@qlu.edu.cn (T.L.); 2 EnviroCORE, Dargan Research Centre, South East Technological University, Carlow Campus, R93 V960 Carlow, Ireland; mutian.wang@postgrad.setu.ie

**Keywords:** hydrocarbon series of petroleum, soil, ecotoxicity, earthworms, metabolism, assessment

## Abstract

**Background/Objectives:** Petroleum contamination in soil exerts toxic effects on earthworms (*Eisenia fetida*) through non-polar narcotic mechanisms. However, the specific toxicities of individual petroleum components remain insufficiently understood. **Methods:** This study investigates the effects of four petroleum components—saturated hydrocarbons, aromatic hydrocarbons, resins, and asphaltenes—on earthworms in artificially contaminated soil, utilizing a combination of biochemical biomarker analysis and metabolomics to uncover the underlying molecular mechanisms. **Results:** The results revealed that aromatic hydrocarbons are the most toxic fraction, with EC50 concentrations significantly lower than those of other petroleum fractions. All tested fractions triggered notable metabolic disturbances and immune responses in earthworms after 7 days of exposure, as evidenced by significant changes in metabolite abundance within critical pathways such as arginine synthesis, a-linolenic acid metabolism, and the pentose phosphate pathway. According to the KEGG pathway analysis, saturated hydrocarbon fractions induced marked changes in glycerophospholipid metabolism, and arginine and proline metabolism pathways, contributing to the stabilization of the protein structure and membrane integrity. Aromatic hydrocarbon fractions disrupted the arachidonic acid metabolic pathway, leading to increased myotube production and enhanced immune defense mechanisms. The TCA cycle and riboflavin metabolic pathway were significantly altered during exposure to the colloidal fraction, affecting energy production and cellular respiration. The asphaltene fraction significantly impacted glycolysis, accelerating energy cycling to meet stress-induced increases in energy demands. **Conclusions:** Aromatic hydrocarbons accounted for the highest level of toxicity among the four components in petroleum-contaminated soils. However, the contributions of other fractions to overall toxicity should not be ignored, as each fraction uniquely affects key metabolic pathways and biological functions. These findings emphasize the importance of monitoring metabolic perturbations caused by petroleum components in non-target organisms such as earthworms. They also reveal the specificity of the toxic metabolic effects of different petroleum components on earthworms.

## 1. Introduction

Petroleum is a complex mixture composed of thousands of distinct components. During crude coil extraction and transportation, released petroleum can migrate, diffuse, and accumulate in soils surrounding oil wells, posing substantial ecological risks [1]. Soil, as a primary reservoir for terrestrial organic matter, serves as a sink for hydrophobic organic pollutants. Petroleum enters soil ecosystems through various pathways, including atmospheric deposition, wastewater irrigation, and the application of biosolids [2]. While total petroleum hydrocarbon (TPH) content is often used to assess contamination levels, it does not accurately reflect the specific toxicity or hazards posed to the environment [3]. Chromatographic methods have categorized petroleum into four key fractions: saturated hydrocarbons, aromatic hydrocarbons, resins, and asphaltenes. However, the toxicity mechanisms of these individual petroleum components and their mixtures on soil organisms remain poorly understood.

Earthworms are critical invertebrates within soil ecosystems, playing a key role as ecological indicators. *Eisenia fetida* is the most widely used species in eco-toxicological investigations as a valuable bio-indicator for monitoring soil contamination, due to its broad dietary preferences, short life cycle, rapid reproduction, and strong regeneration ability [4,5]. Metabolomics studies have revealed that exposure to heavy metals, industrial pollutants, and pesticides can disrupt the metabolic profiles of earthworms. Recent research into petroleum toxicity suggests that hydrocarbons reduce the activity of biomarker enzymes such as peroxidase, whereas increase oxidative stress markers such as malondialdehyde (MDA). However, whether these enzymatic changes directly influence downstream metabolic processes remain unclear. Metabolomics, which provides a quantitative understanding of metabolites within integrated biological systems, is increasingly used to investigate the environmental impacts of pollutants. In the assessment of the biological toxicity of petroleum contaminants in soil matrices, Hou et al. [6] investigated the metabolic differences of petroleum pollutants in various soils and revealed that both petroleum concentration and its carbon composition differentially influenced the metabolism of earthworms. Simpson et al. [7] combined traditional ecological toxicity endpoints for earthworms with ^1^H NMR-based metabolomics to compare the responses of earthworms exposed to petroleum hydrocarbons in aged contaminated agricultural soils, which concluded the differences in toxicity between short-term and long-term exposures. However, the potential toxicity mechanisms of earthworms exposure to different petroleum fractions remain unclear. Environmental metabolomics aims to assess individual, population, and ecological health by identifying metabolic biomarkers and observing functional changes induced by pollutant exposure at various biological levels. This approach aligns with adverse outcome pathways (AOP) models, which connect molecular-level disruptions to population-level effects. Griffith et al. (2018) argued that metabolomics can serve as a valuable tool for elucidating modes of action (MOA) related to toxic effects and refining AOPs [8]. However, applying metabolomics in systemic toxicology is associated with challenges, such as validating and predicting biomarkers, linking metabolic changes to critical outcomes, and elucidating species-specific metabolic pathways. Furthermore, the relationship between molecular structure and metabolic effects has been insufficiently explored, although structure-activity relationships (SARS) could provide valuable insights for predicting toxicity and developing biomarkers. This study focuses on how exposure to structurally diverse petroleum components affects the metabolic profiles of earthworms (*Eisenia fetida*), shedding light on the structural basis of their toxic effects on metabolism in organisms.

By exposing earthworms to artificially contaminated soil for 7 days, this study provides comprehensive insights into the toxic effects, oxidative stress, and metabolic disruptions caused by petroleum fractions on earthworms. The objectives of this study were as follows: (1) To determine the median effect concentration (EC50) of petroleum fractions on earthworms in soil though exposure tests. (2) To evaluate the effects of exposure to petroleum fractions on the enzyme activities of earthworms. (3) To investigate the impacts of petroleum exposure on earthworm metabolites and uncover the molecular mechanisms underlying their toxicity to earthworms. This study offers new insights into the effects and mechanisms of toxicity of petroleum components in soil environments and provides further data to more accurately assess ecological risks for terrestrial ecosystems.

## 2. Materials and Procedures

### 2.1. Analysis Method of the Petroleum

The separation of resins and asphaltenes was performed using the SARA (Saturates, Aromatics, Resins, Asphaltenes) four-component separation method, following the extraction NB/SH/T 0509-2010 protocol. Saturated and aromatic hydrocarbons were obtained from McLean Biochemical Technology Co., Ltd., Shanghai, China, in equimolar ratios using representative substances. Detailed descriptions of the specific pollutants used in the mixtures are provided in Supplementary Material ST1.

### 2.2. Earthworm for the Experiment

Adult earthworms (*E. fetida*) were procured from the Jiangsu Earthworm Breeding Base. Healthy specimens of adult earthworms with an average weight of 0.35 g and uniform age, featuring a distinct clitellum, were selected for the experiments. The methodology followed the OECD 207 guidelines. Before exposure, the earthworms were acclimatized for 1 week in contaminant-free soil at 20 ± 1 °C under a 12:12-h light/dark cycle with 65% light exposure.

### 2.3. Toxicity Test Procedure

The experimental soil was collected from the Shandong Academy of Sciences’s yard (latitude 36°39′ N, longitude 117°15′ E) at a 10- to 30-cm depth. Before being used in the experiment, the soil was air-dried, milled, and sieved using a 10-mesh sieve. The soil was classified as silty loam with a pH of 7.19. In total, 10 mL dichloromethane was used to uniformly mix the pollutants evenly into the soil, followed by vigorous stirring with a stainless-steel spatula to ensure even distribution. The mixture was left in a fume hood for 24 h to allow complete organic solvent evaporation. For each treatment, 10 earthworms were placed into a 600-mL beaker containing the contaminated soil for cultivation. The beakers were kept in a climate-controlled chamber set at 20 ± 1 °C with photoperiod and 79 ± 1% ambient humidity and in total darkness. The earthworms were fed oats, and soil moisture was maintained at 15%. Control groups were treated under identical conditions. Each treatment was performed in triplicate. After 7 days of exposure, surviving earthworms were counted, rinsed with Milli-Q water, dried with paper towels [9], and weighed. They were then rapidly frozen in liquid nitrogen and stored at −80 °C until further analysis.

### 2.4. Acute Toxicity Analysis

Survival data for earthworms exposed to varying pollutant concentrations were recorded to calculate the median effect concentration (EC50) for each of the four petroleum fractions. Statistical analyses and graph generation were performed using SPSS (IBM Corp., Armonk, NY, USA) and Origin software (SR1b9.5.1.195).

### 2.5. Biochemical Indicators and Analysis

Following the 7-day exposure period, one earthworm from each of the three replicates per treatment group was collected. The samples were mixed, freeze-dried, homogenized, and powdered to obtain a uniform earthworm tissue. The activities of superoxide dismutase (SOD) and levels of MDA were measured according to the protocols provided by the manufacturer of the BIYUNTIAN test kits. Details of these procedures are described in Supplementary Material ST2. One-way ANOVA in SPSS software (IBM Corp., Armonk, NY, USA. IBM SPSS Statistics 25.0) was tested to assess significant differences(*p* < 0.05) and plotted using Origin software (SR1b9.5.1.195).

### 2.6. Metabolite Analysis

Metabolites in earthworms exposed to sublethal concentrations of petroleum fractions (saturated hydrocarbons, aromatic hydrocarbons, resins, and asphaltenes) through liquid chromatography-mass spectrometry (LC-MS). The full details of metabolite extraction and LC-MS/mass spectrometry conditions are outlined in Supplementary Material ST3. Principal component analysis was conducted to provide an overview of clustering among the groups. To further differentiate between treatment groups, partial least squares discriminant analysis (PLS-DA) was used to identify significantly altered metabolites between the groups. Criteria for significance included a Variable Importance in Projection (VIP) score of >1 and *p* < 0.05. To assess hierarchical differences, multi-block PLS hierarchical cluster analysis was performed using SIMCA. This analysis applied multi-block PLS dimensionality reduction across all samples, grouping by treatment type and disregarding dosing effects. Cluster distances were calculated using the Ward method. Results were visualized in dendrograms, sorted by cluster size [8].

## 3. Results

### 3.1. Effects of Exposure to Petroleum Components on Earthworm Survival

**Figure 1:**
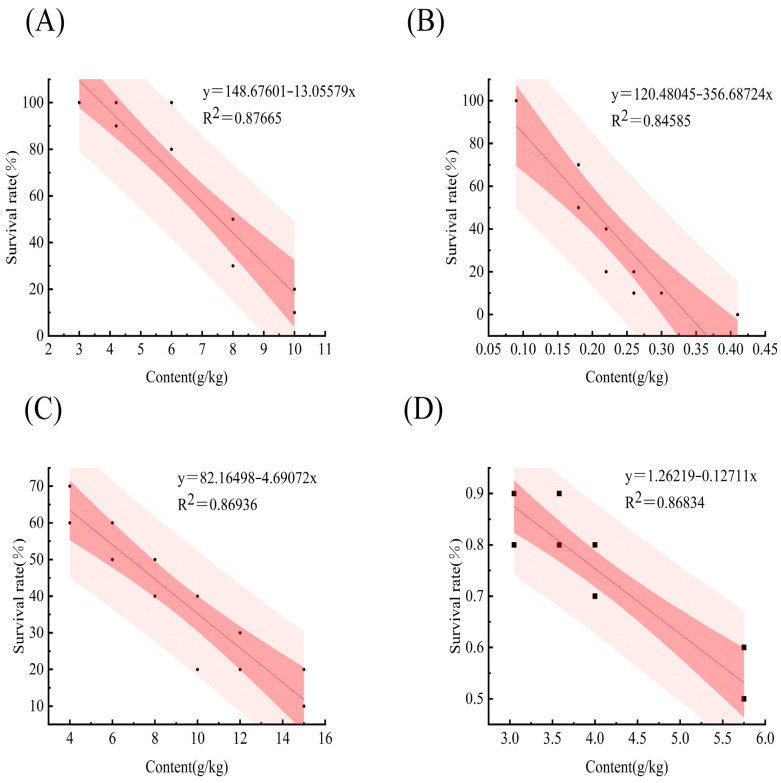
Earthworm survival dose-effect diagram. (**A**–**D**) represent the survival rate of earthworms exposed to saturated hydrocarbons, aromatic hydrocarbons, colloidal fractions, and asphaltene fractions.

The impact of exposure to different petroleum components on earthworm survival was evaluated using the median effect concentration (EC50) from toxicity tests [10]. A lower EC50 value signifies higher toxicity. A single toxicity test was conducted on earthworms exposed to varying concentrations of saturated hydrocarbons, aromatic hydrocarbons, colloidal fractions, and asphaltenes. The concentration-response relationships were analyzed, and the EC50 values for the four petroleum components in soil were determined (Figure 1). The data suggest the following ranking of soil toxicity (EC50 in g/kg): aromatic hydrocarbons (0.2) > asphaltenes (6) > resins (6.86) > saturated hydrocarbons (7.53).

### 3.2. Evidence from Biomarkers

**Figure 2:**
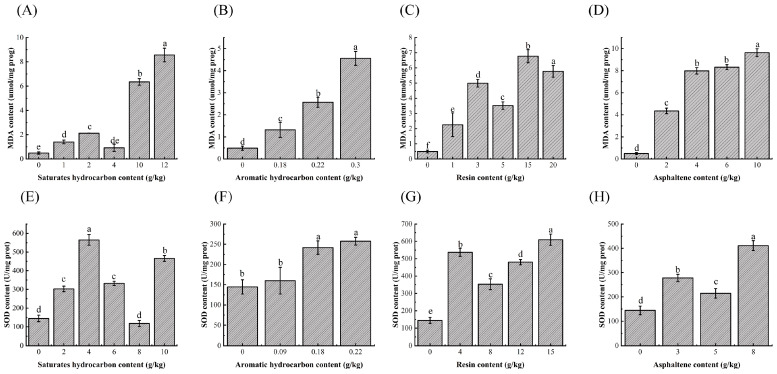
(**A**–**D**) represent changes in biomarker MDA and SOD content in earthworms after exposure to the four petroleum components. (**E**–**H**) indicate the change in SOD content. (**A**–**H**) Bars represent changes in earthworms due to saturated hydrocarbons, aromatic hydrocarbons, resins, and asphaltenes as contaminant staining. The x-axis indicates treatment concentrations, and the y-axis represents the enzyme activity normalized to protein content (U mol/mg prot^−1^). Different letters denote significant differences between treatments (ANOVA, Tukey test, *p* < 0.05).

To assess the ecological toxicity of petroleum components, we measured changes in oxidative stress biomarkers. As shown in Figure 2, MDA levels and oxidative stress products SOD activity were significantly higher in contaminated earthworms than in control groups (*p* < 0.05, Figure 2). Specifically, when the concentrations of saturated hydrocarbons, resins, and asphaltenes in the soil reached 1 g/kg, MDA levels increased significantly, indicating that oxidative damage occurred due to the conversion of superoxide anions (O_2−_) to hydrogen peroxide (H_2_O_2_). This suggested that the earthworms’ antioxidant systems could not effectively counterbalance the additional oxidative stress. Remarkably, even at a lower concentration of 0.18 g/kg, aromatic hydrocarbons caused significant damage to earthworms, with MDA levels reaching 1.32 U/g protein. MDA levels were found to vary based on exposure dosage, revealing a nonlinear dose–response relationship.

### 3.3. Metabolomics Evidence

**Figure 3:**
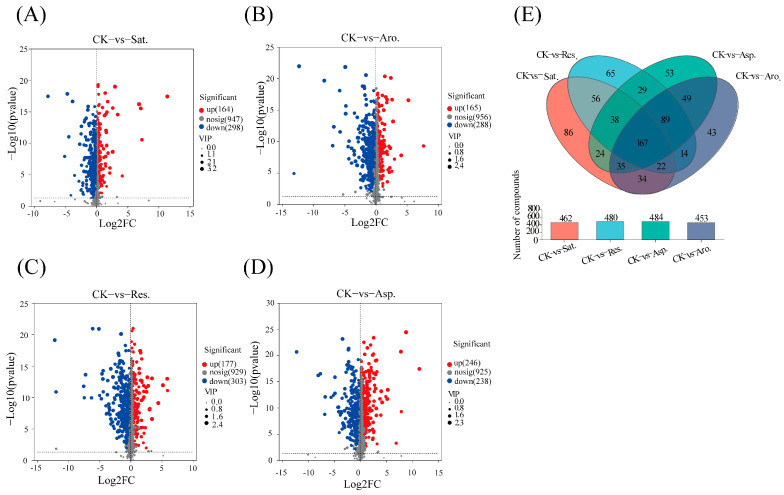
Volcano plots of metabolite profiles (**A**: CK vs. Sat., **B**: CK vs. Aro., **C**: CK vs. Res., **D**: CK vs. Asp.) and Venn diagram of differential metabolites (**E**) in earthworms.(where CK is the control group; Sat. is the saturated hydrocarbon component exposure group; Aro. is the aromatic hydrocarbon component exposure group; Res. is the colloidal component exposure group; and Asp. is the asphaltene component exposure group, and these labels are used below).

**Figure 4:**
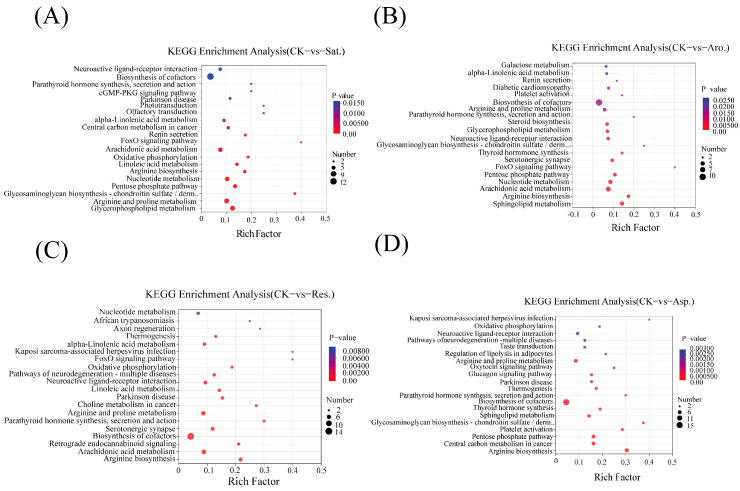
KEGG pathway enrichment of differential metabolites across treatment groups. (**A**–**D**) represent KEGG pathway analysis results for each exposure group, with significant pathways indicated for VIP ≥ 1, *p* ≤ 0.05.

Metabolomic analysis (non-targeted metabolomics) revealed significant differences in the metabolite profiles of earthworms exposed to various petroleum components compared to the control group. Figure 3 presents a volcano plot (Figure 3) showing the distribution of metabolites between the experimental groups and control group. A Venn diagram was constructed for metabolites with VIP > 1 and *p* < 0.05. The results indicated 462, 453, 480, and 484 metabolites for comparisons between the control (CK) and the saturated hydrocarbons, aromatic hydrocarbons, resins, and asphaltenes, respectively. A total of 34 differential metabolites (DMs) were identified across the different exposure groups (CK vs. Sat. vs. Aro. vs. Res. vs. Asp.). The KEGG pathway enrichment analysis highlighted significant metabolic alterations in exposed earthworms. For example, exposure to saturated hydrocarbons predominantly affected pathways related to cofactor biosynthesis, arachidonic acid metabolism, nucleotide metabolism, pentose phosphate pathway, arginine and proline metabolism, and glycerophospholipid metabolism (Figure 4A). By contrast, exposure to aromatic hydrocarbons enriched pathways linked to cofactor biosynthesis, nucleotide metabolism, arachidonic acid metabolism, and sphingolipid metabolism compared with CK (Figure 4B). Exposure to resins primarily influenced pathways involved in neuroactive ligand–receptor interactions, arginine and proline metabolism, serotonergic synapses, cofactor biosynthesis, arachidonic acid metabolism, and arginine biosynthesis (Figure 4C).Compared with CK, exposure to asphaltenes enriched pathways involved in neuroactive ligand–receptor interactions, arginine and proline metabolism, cofactor biosynthesis, sphingolipid metabolism, pentose phosphate pathway, central carbon metabolism in cancer, and arginine biosynthesis (Figure 4D). Common KEGG pathways significantly enriched across the groups included cofactor biosynthesis and arachidonic acid metabolism, indicating consistent alterations in energy production and inflammatory responses.

Figures S1 and S2 illustrate the differences in metabolites across earthworms exposed to various petroleum components. The four petroleum fractions predominantly influenced amino acid metabolism and lipid metabolism. Among the components, saturated hydrocarbons and aromatic hydrocarbons had the most significant impact on lipid metabolism and amino acid metabolism. By contrast, resins primarily affected cofactor and vitamin metabolism, while asphaltenes notably impacted amino acid metabolism and carbohydrate metabolism. Supplementary Material Figure S3 provides a breakdown of the compound categories for DMs compared with the control group. The DMs across the different exposure groups were predominantly phospholipids, nucleotides, amino acids, and fatty acids. Notably, each petroleum component—saturated hydrocarbons, aromatic hydrocarbons, and asphaltenes—featured unique DMs compared with the control, such as lactams in saturated hydrocarbons, oligosaccharides in aromatic hydrocarbons, and carboxylic compounds in asphaltenes.

**Figure 5:**
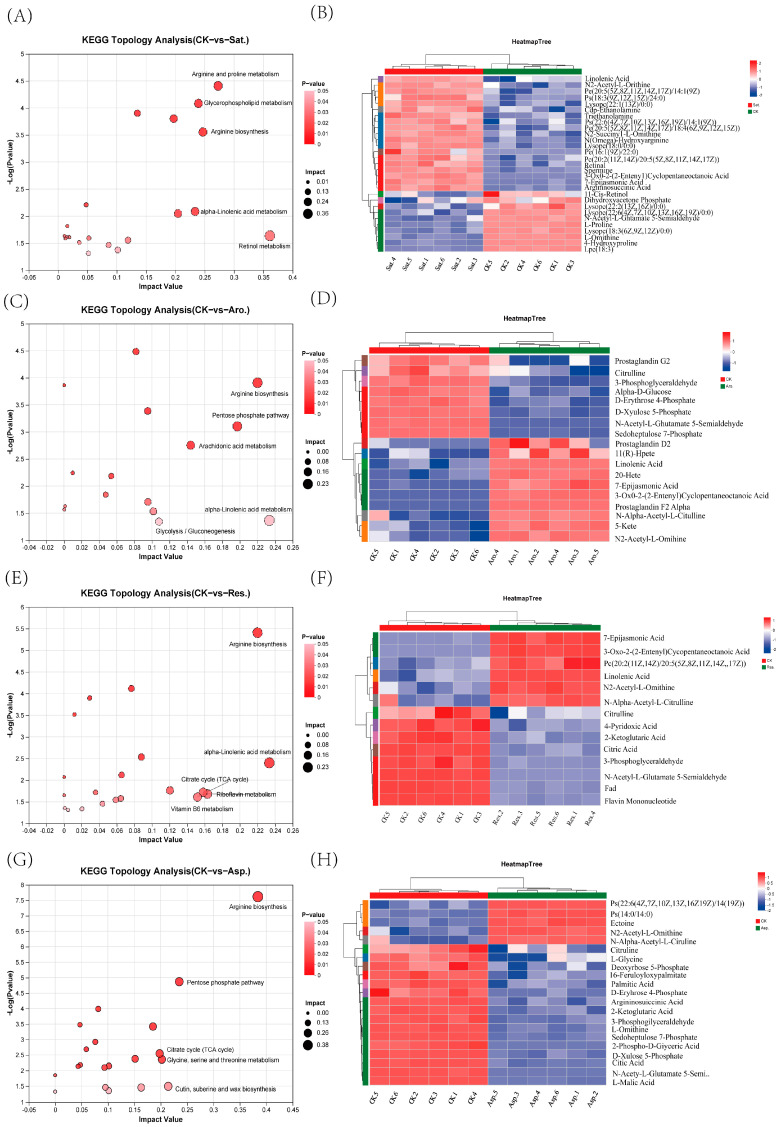
Metabolomic analysis of DMs across different petroleum component exposure groups compared with the control group. Pathway analyses and heatmaps illustrate upregulated (red) and downregulated (blue) metabolites. The exposure groups analyzed were saturated hydrocarbons (**A**,**B**), aromatic hydrocarbons (**C**,**D**), glycosyl components (**E**,**F**), and asphaltenes (**G**,**H**) (*p* < 0.05). Significant pathways with *p* < 0.05 are depicted.

**Figure 6:**
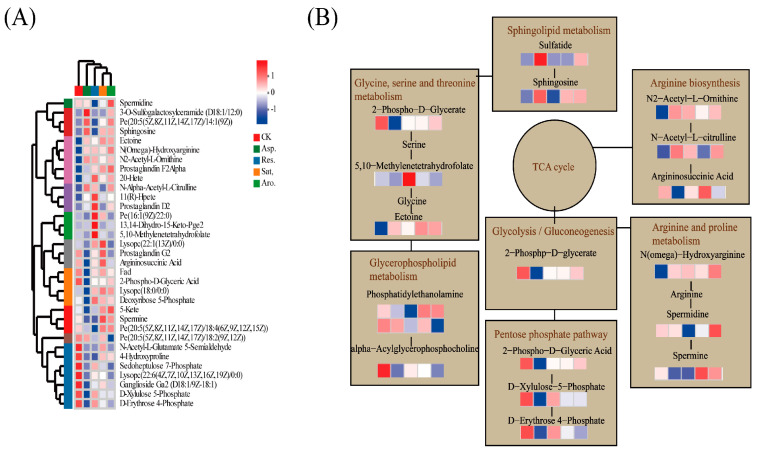
Heatmap of hierarchical clustering of potentially discriminating metabolites obtained from the treatment groups (CK, Sat., Aro., Res., and Asp.) (**A**). Values of the Pearson correlation coefficient matrix for various biological indicators of DMs (*p* < 0.05, VIP > 1) (**B**).The relative amino acid content is represented by a color scale from blue (minimum) to red (maximum). The groups are labeled as CK (control), Asphaltene fraction, Resin fraction, Saturated hydrocarbon fraction, and Aromatic hydrocarbon fraction.

Topological analysis of the KEGG pathways revealed the top five most significant pathways for each petroleum component exposure group, as well as their corresponding DMs (Figure 5). In the group exposed to saturated hydrocarbons, five significant pathways were identified based on pathway impact values (PIV > 0.05), including arginine and proline metabolism, glycerophospholipid metabolism, arginine biosynthesis, alpha-linolenic acid metabolism, and retinol metabolism (Figure 5A). Among these five pathways, 29 metabolites were significantly altered (Figure 5B). In the group exposed to aromatic hydrocarbon components, five significant pathways were determined based on PIV > 0.05, namely aArginine biosynthesis, pentose phosphate pathway, arachidonic acid metabolism, alpha-linolenic acid metabolism, and retinol metabolism (Figure 5C). Within these five pathways, 18 metabolites exhibited significant changes (Figure 5D). In the group exposed to glycosyl components, five key pathways were identified based on PIV > 0.05, namely arginine biosynthesis, alpha-linolenic acid metabolism, citrate cycle (tricarboxylic acid (TCA) cycle), riboflavin metabolism, and pentose phosphate pathway (Figure 5E). In this group, 13 metabolites were significantly affected (Figure 5F). In the group exposed to asphaltene components, five crucial pathways were identified based on PIV > 0.05, which include arginine biosynthesis; pentose phosphate pathway; cutin, suberine, and wax biosynthesis; alpha-linolenic acid metabolism, and glycolysis/gluconeogenesis (Figure 5G). Among these pathways, 16 metabolites were significantly affected (Figure 5H). Supplementary Material Figure S4 provides a comprehensive overview of the significant pathways associated with the upregulated and downregulated metabolites for each petroleum component compared with the control group. Metabolite enrichment analysis (6A) was performed on the DMs in the significant metabolic pathways identified in Figure 5. Figure 6B presents the integrated KEGG metabolic pathway network of key metabolites in the different component exposure groups. This analysis provided a deeper understanding of the metabolic disruptions caused by petroleum component exposure.

**Figure 7:**
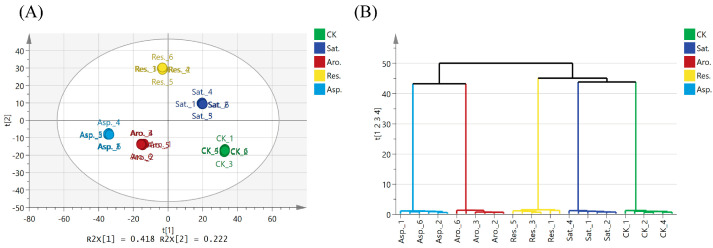
MB-PLS-DA of all data as a function of treatment. (**A**) Score plot showing different treatment groups. (**B**) Dendrogram describing the MB-PLS-HCA of treatment groups, with hierarchal clustering calculated using Ward’s methods and sorted by size.

PLS analysis was used to assess the metabolic phenotype differences among the treatment groups (Figure 7A). Over the 7-day exposure period, a clear separation was observed between the treatment and control groups, indicating substantial metabolic alterations in the earthworms due to petroleum exposure. The multi-block partial least squares hierarchical cluster analysis (MB-PLS-HCA) (Figure 7B) further confirmed these differences, highlighting distinct clustering patterns between the treatment groups.

## 4. Discussion

### 4.1. Effects of Different Components of Petroleum Hydrocarbons on Earthworm Survival

The results of a 7-day exposure experiment on earthworms indicate that in soil media, the toxicity of petroleum fraction components follows the order: aromatic hydrocarbons > asphaltenes > resins > saturates. Similarly, a toxicity test on sea urchin embryos conducted by Diego Rial et al. [11] in aquatic media showed that the toxicity of different petroleum components (µL L^−1^) follows the order: saturates (165.8–242.3) < polar compounds (87.1–115.7) < aromatic hydrocarbons (20.5–34.6), revealing the same relative toxicity pattern for petroleum fractions. The significantly lower toxicity concentrations in water media compared to soil suggest that petroleum components in aquatic environments have a more pronounced impact on organisms, possibly because their absorbable properties by aquatic species. As polar compounds (resins and asphaltenes) contribute more to the toxicity of crude oil as weathering progresses, they are generally more toxic than saturated hydrocarbon fraction. Singh [12] reported that aromatic hydrocarbons and heavy asphaltenes exhibit similar toxicity, and for different oil categories tested using microalgae, their toxicity was greater than that of aliphatic compounds. These findings underscore the need for further research utilizing techniques beyond traditional GC-MS to identify the specific polar compounds or compound groups, as well as to establish causal relationships between these compounds and the observed toxicity.

### 4.2. Effects of Different Components of Petroleum Hydrocarbons on Biomarkers in Earthworms

Exposure to pollutants, including petroleum hydrocarbons, induces oxidative stress in earthworms. This stress arises when the production of reactive oxygen species (ROS) surpasses the organism’s antioxidant defense capacity, leading to cellular damage. Oxidative stress manifests as lipid peroxidation, protein and DNA damage, and apoptosis in earthworms [13,14]. To counteract this, the antioxidant enzyme system in earthworms, including SOD, catalase (CAT), and glutathione peroxidase (GPX), is activated to reduce the accumulation of ROS, such as hydrogen peroxide (H_2_O_2_), thus serving a self-defense role. SOD catalyzes the dismutation of superoxide anion (O_2−_) to H_2_O_2_ and O_2_, thus serving as the first line of defense against ROS. SOD, CAT, GPX and MDA are key antioxidant enzymes that reduce cellular oxidative damage and are commonly used as biomarkers of pollutant exposure-induced oxidative damage. MDA, a lipid electrophilic agent produced during lipid peroxidation, is a common biomarker of oxidative damage, as it directly reflects the severity of lipid peroxidation and membrane integrity disruption. Elevated MDA levels can also lead to protein and nucleic acid aggregation, inducing cytotoxicity [15]. Shi et al. (2020) demonstrated that phenanthrene exposure at or above 5 mg/kg in soil significantly increases MDA levels [14,16]. Similarly, a significant elevation in MDA content was observed in earthworms exposed to petroleum-contaminated environments for 7 days [17].

The SOD activity in earthworms was the lowest in soils contaminated with aromatic hydrocarbons. This is likely due to the high toxicity of aromatic components, which disrupt earthworm growth and metabolic processes. Under high toxic stress, when ROS production exceeds the detoxification capacity of earthworms, functional membranes and enzyme systems within the cells are damaged, leading to metabolic disturbances and significantly reduced SOD activity, which then triggers lipid peroxidation. Conversely, exposure to less toxic components such as saturated hydrocarbons, resins, and asphaltenes stimulates the earthworms’ defense mechanisms, which involves stimulation and acceleration of resistant physiological activities of earthworms. In these cases, SOD activity increases, enhancing ROS degradation and mitigating oxidative damage. Therefore, earthworms exposed to aromatic hydrocarbons exhibited significantly lower SOD activity in total petroleum hydrocarbon (TPH)-contaminated soil than in soil contaminated with other hydrocarbon components.

### 4.3. Effects of Different Components of Petroleum Hydrocarbons on Earthworm Metabolites

This study systematically evaluated the ecological toxicity of petroleum hydrocarbons on earthworms using non-targeted metabolomics techniques. Exposure to petroleum components altered oxidative stress indicators and significantly disrupted the metabolic phenotype of earthworms.

#### 4.3.1. Action Mechanisms by Different Components

The metabolomic analysis of earthworms exposed to EC50 levels of petroleum components (Figure 5) highlighted significant disruptions in key metabolic pathways, as evidenced through the KEGG topology analysis. The most affected pathways were lipid metabolism and amino acid metabolism.

Significant pathways exhibiting upregulated and downregulated of differentially expressed metabolites in the four component exposure groups were analyzed in comparison with the control group (Supplementary Figure S4). The pentose phosphate pathway, TCA cycle, and glycolytic pathway showed notable upregulation across treatment groups (Supplementary Figure S4). Metabolic pathways are regulated in response to oxidative stimuli, with the pentose phosphate pathway commonly upregulated to enhance or maintain cellular reducing capacity via NADPH production, mitigating oxidative damage. Three enzymes of its pathway exhibited varying sensitivities to different pollutants [18,19]. The glycolytic pathway provides pyruvate for oxidative metabolism, and in addition to its role in ATP production, produces metabolic intermediates for various cellular functions [20]. The pentose phosphate and glycolytic pathways are the primary routes for cellular production of NADPH, a potent reductant essential for maintaining cellular redox balance [20]. Notably, in the presence of contaminating gel components, substances such as vitamin B6 and riboflavin, are upregulated, which suggests enhanced antioxidant defense under stress.

All the four petroleum components suppressed α-linolenic acid metabolism and glycerophospholipid metabolism, primarily due to oxidative stress-induced ROS accumulation [21]. To overcome this oxidative damage, α-linolenic acid and glycerophospholipids exert antioxidant functions. α-Linolenic acid is primarily converted into anti-inflammatory substances and serves as an antioxidant [16]. When alfalfa seedlings were exposed to vanadium, allene oxide cyclase (AOC) and acetyl-CoA acyltransferase (fadA) genes in α-linolenic acid metabolism were significantly downregulated [22]. Glycerophospholipids are essential for cell membrane integrity. Petroleum exposure disrupts this pathway, leading to impaired cell membrane stability and function. In summary, oxidative stress from petroleum hydrocarbons disrupted metabolic pathways, including glycolysis, TCA cycle, amino acid metabolism, and lipid metabolism, contributing to neurotoxicity, inflammation, oxidative damage, and dysregulation of osmotic balance and energy metabolism.

#### 4.3.2. Biochemical Disturbances Induced by Petroleum Components

Amino acids essential for protein synthesis, signal transduction, energy metabolism, and oxidative stress responses [23]. According to some studies, amino acids such as leucine and valine can serve as potential biomarkers of responses of earthworm to PAH exposure [24]. Notably, exposure of earthworms to four petroleum components significantly altered the arginine synthesis pathway, particularly involving compounds such as L-argininosuccinic acid. Li et al. (2023) found that altered metabolites primarily participated in glycerophospholipid metabolism, arginine and proline metabolism, and arginine biosynthesis in mice exposed to cadmium toxicity [25,26]. Arginine is crucial for enhancing the antioxidant capacity of organisms through a mechanism similar to that of antioxidant enzymes [27]. Proline (Pro), a small-molecular-weight soluble amino acid with osmoregulatory properties, is a major animal collagen component and interacts with proteins to form a hydrophobic backbone, thereby thus stabilizing the protein structure and the biological membrane integrity. Proline is a crucial player in endogenous reactive oxygen scavengers and forms a protective system to augment biological resistance [28,29]. Arginine and proline metabolism increased in the group exposed to saturated hydrocarbons, whereas decreased in the group exposed to asphaltenes. Arginine and proline metabolism was a non-significantly different pathway in the remaining two groups (Supplementary Figure S4).

In earthworms, asphaltene components significantly enriched differentially expressed metabolites in the glycine, serine, and threonine metabolic pathways (Figure 5G). Both AhR-dependent and independent mechanisms are involved in the toxicity of weathered light asphaltene in zebrafish [30], By using ^1^H NMR to identify metabolites, four affected pathways were identified, namely glycolytic, serine, and threonine metabolic pathways, as well as pathways involved in the synthesis and metabolism of other amino acids [31]. Furthermore, pyrene—an archetypal nonpolar organic compound—exerts its nonspecific toxic effects by increasing the production of various amino acids such as lysine, which suggests impairment in carbohydrate metabolism is correlated with increased fatty acid metabolism and changes in TCA cycle intermediates [32]. Metabolic-level differences in energy transfer molecules, carbohydrates, organic acids, osmoregulatory agents, and amino acids were observed between earthworms in uncontaminated and PHC-contaminated soils [7].

Fatty acids are fundamental components of metabolic fuel and lipids. When d-glucose is absent, fatty acid oxidation in muscle serves as a primary energy source. Arachidonic acid metabolism was significantly downregulated following exposure to aromatic hydrocarbons and resins (Supplementary Figure S4). Arachidonic acid metabolism promotes myonuclei proliferation and myotube growth during muscle development [33], which possibly serve as a defensive response against hydrocarbon exposure in earthworms. Consequently, exposure to aromatic compounds and resins compounds can inhibit fatty acid biosynthesis, thereby suppressing growth.

In challenging environments, earthworms require sufficient energy to support normal physiological activities and sustain growth [34]. Glycolysis/gluconeogenesis and the TCA cycle are the primary mechanisms for energy production and supply in living organisms. Glycolysis generates pyruvic acid, which is essential for fueling the TCA cycle [35]. Within the TCA cycle, pyruvic acid undergoes aerobic reactions, releasing additional energy. Levels of glycolytic intermediates, such as 2-phospho-D-glycerate, were significantly downregulated after 7 days of exposure to all petroleum hydrocarbon components. This observation suggests that earthworms activate their glycolytic pathways more intensively to compensate for energy demands during pollutant exposure [36]. In conclusion, the reduction in glycolytic intermediate metabolites indicates a compensatory increase in energy production to meet heightened physiological requirements. This adaptation highlights one of the key survival mechanisms earthworms employ when enduring the stress of long-term pollutant exposure.

Glycerophospholipids re major structural lipids providing stability, fluidity, and permeability to mammalian membranes. They are involved in various cellular processes, including signal transduction, vesicle transport, and cell division [25]. Exposure to all four petroleum components reduced glycerophospholipid content in the groups exposed to saturated hydrocarbons and asphaltenes, whereas the groups exposed to the other two components exhibited upregulatory and downregulatory effects on glycerophospholipids (Supplementary Figure S4). In mice, cadmium toxicity is associated with an imbalance in glycerophospholipid metabolism [26,37].

Sphingosine, phytosphingosine, and dihydrosphingosine are produced in simple sphingolipid metabolism. Sphingolipids undergo dehydration with long-chain fatty acids to form ceramides, which are cell membrane components. Compared to the control, exposure to aromatic hydrocarbons and asphaltene components weakened the sphingolipid metabolic pathway (Figure S4), but the sphingolipid content increased relative to the control group (Figure 6B).The phenomenon could be a result of defenses of the organism against pollutants. There exists a dynamic network regulation between sphingolipid content and other metabolites [38]. The study by Li et al. indicates that the biosynthetic network of lipids, including phospholipids, glycerophospholipids, sphingolipids, and eicosanoids, is essential in animals, and these lipids are interconnected through various biochemical reactions [39]. Hou et al. identified biomarkers associated with cellular uptake, stress responses, and membrane disruption in earthworms from different oilfields [6]. Exposure to petroleum components heightened sphingolipid metabolic pathways, which critically involved in lipid regulation and lipid peroxidation [23]. In fish, crude oil and chemical dispersants downregulated DMs enriched in sphingolipid metabolism [21].

In summary, exposure to all petroleum fractions triggered immune defense mechanisms and influenced exogenous metabolism in earthworms compared with the control group, though distinct metabolomic responses were observed. Saturated hydrocarbon fraction exposure helped stabilize the protein structure and maintain biofilm integrity by promoting the metabolic pathways of arginine and proline. By contrast, exposure to the aromatic hydrocarbon fraction increased arachidonic acid metabolites, enhancing immune defense. Exposure to resins and asphaltenes impaired energy cycling. The unique chemical properties of the contaminants may contribute to their different metabolomic responses.

#### 4.3.3. Structure-Activity Relationships of Petroleum Components

The MB-PLS-DA score plots for all samples (Figure 7A), organized by treatment, further support the hypothesis that different petroleum components induce distinct metabolic profiles in earthworms. The MB-PLS-HCA (Figure 7B) further refines findings, highlighting clear clusters. Notably, a significant distinction exists between the cluster of saturated hydrocarbons and that of aromatic hydrocarbons. A subsequent sub-cluster emphasizes the similarity between aromatic hydrocarbons and asphaltenes, as well as between saturated hydrocarbons and the control group. Overall, the MB-PLS-HCA reveals good aggregation among the treatment groups, suggesting that earthworms exhibit specificity in their metabolic responses to various petroleum components. Griffith et al. [8] explored the structure-activity relationships of various herbicides, showing that structural differences among herbicides lead to distinct clustering based on their chemical compositions.

These findings are further supported by several considerations. The toxicity of saturated hydrocarbons is lower than that of the other exposure groups, likely because many saturated hydrocarbons are linear aliphatic compounds, which are more easily utilized and metabolized by earthworms after ingestion. The toxicity pattern of resin-like hydrocarbons closely mirrors that of saturated hydrocarbons, likely because of the presence of linear alkane constituents in the resin components. Using the SARA separation method, Boukir et al. [40] obtained resins and analyzed their chemical composition and structural characteristics through Fourier-transform infrared spectroscopy. Their analysis revealed the presence of linear and branched alkanes, ranging from C10–C30, along with n-alkanes, branched alkanes, and cycloalkanes.

The similarity in clustering between asphaltenes and aromatic hydrocarbons is attributable to the high content of aromatic compounds in asphaltenes. The toxicity of diluted bitumen is believed to be linked to PAHs, which bind and activate the aryl hydrocarbon receptor [41]. The toxicity of aromatic hydrocarbons is driven by benzene ring accumulation, which enhances their ability to form hydrogen bonds with biomolecules. This interaction leads to conjugate addition reactions with nucleophilic groups in proteins and nucleic acids (RNA and DNA), thereby resulting in irreversible covalent bonds and causing protein function loss and mutagenic DNA damage. Ivanei et al. [42] found that the higher content of aromatic hydrocarbons, heteroatoms, polar substances, and hydroxyl groups in heptane asphaltenes contributes to their instability. Moreover, the structural similarity between aromatic components, resin-like components, and asphaltenes is more pronounced when compared to saturated hydrocarbons.

## 5. Conclusions

This study comprehensively assessed the toxic effects, oxidative stress responses, and metabolic mechanisms of petroleum hydrocarbon exposure on earthworms. The results demonstrated that in soil matrices, the toxicity of petroleum hydrocarbons followed the same order: aromatic hydrocarbons > resins > asphaltenes > saturated hydrocarbons. Exposure to petroleum hydrocarbons induced oxidative stress in earthworms, leading to increased lipid peroxidation and decreased antioxidant capacity. Petroleum hydrocarbon exposure caused significant alterations in metabolites, with disruptions observed in glycolysis, the TCA cycle, amino acid metabolism, and lipid metabolism. These changes were validated through significantly enriched KEGG pathways. Saturated hydrocarbons exposure of the earthworms resulted in notable changes in glycerophospholipid metabolism and arginine/proline metabolism pathways.. Exposure to aromatic hydrocarbons led to a downregulation of arachidonic acid metabolism and sphingolipid synthesis. While exposure to resins significantly altered the TCA cycle, arachidonic acid metabolism, and riboflavin metabolism pathways. Exposure to asphaltenes enhanced the TCA cycle and pathways related to glycine, serine, and threonine biosynthesis, but restrained metabolism rates of arginine, proline, and sphingolipid. This study not only elucidates the molecular regulatory mechanisms of earthworm responses to four types of petroleum hydrocarbons but also helps to understand the ecological risk of petroleum hydrocarbons in the environment. Future research may benefit from transcriptomic and proteomic analyses to reveal the differences in earthworm responses to petroleum hydrocarbon stress at the gene and protein levels.

## Data Availability

The original contributions presented in this study are included in the article/Supplementary Materials. Further inquiries can be directed to the corresponding authors.

## Supplementary Materials

The following supporting information can be downloaded at: https://www.mdpi.com/article/10.3390/metabo15020097/s1, Figure S1: Diagram of iPath metabolic pathway analysis; Figure S2: Sankey diagram of the first five differential metabolic pathways of the four components; Figure S3: Individual difference plots for the four fractions; Figure S4: KEGG topology.

## References

[1] Wu B., Guo S., Wang J. (2021). Spatial ecological risk assessment for contaminated soil in oiled fields. J. Hazard. Mater..

[2] Gao Y., Wang L., Zhang X., Shi C., Ma L., Zhang X., Wang G. (2022). Similarities and differences among the responses to three chlorinated organophosphate esters in earthworm: Evidences from biomarkers, transcriptomics and metabolomics. Sci. Total Environ..

[3] Khudur L.S., Gleeson D.B., Ryan M.H., Shahsavari E., Haleyur N., Nugegoda D., Ball A.S. (2018). Implications of co-contamination with aged heavy metals and total petroleum hydrocarbons on natural attenuation and ecotoxicity in Australian soils. Environ. Pollut..

[4] Yadav R., Kumar R., Gupta R.K., Kaur T., Kiran, Kour A., Kaur S., Rajput A. (2023). Heavy metal toxicity in earthworms and its environmental implications: A review. Environ. Adv..

[5] Ferber T., Slaveykova V.I., Sauzet O., Boivin P. (2019). Upward mercury transfer by anecic earthworms in a contaminated soil. Eur. J. Soil Biol..

[6] Hou Z., Mo F., Zhou Q. (2023). Elucidating response mechanisms at the metabolic scale of *Eisenia fetida* in typical oil pollution sites: A native driver in influencing carbon flow. Environ. Pollut..

[7] Whitfield Aslund M., Stephenson G.L., Simpson A.J., Simpson M.J. (2013). Comparison of earthworm responses to petroleum hydrocarbon exposure in aged field contaminated soil using traditional ecotoxicity endpoints and 1H NMR-based metabolomics. Environ. Pollut..

[8] Griffith C.M., Morgan M.A., Dinges M.M., Mathon C., Larive C.K. (2018). Metabolic Profiling of Chloroacetanilide Herbicides in Earthworm Coelomic Fluid Using 1H NMR and GC–MS. J. Proteome Res..

[9] Yang X., Zhang X., Shu X., Gong J., Yang J., Li B., Lin J., Chai Y., Liu J. (2023). The effects of polyethylene microplastics on the growth, reproduction, metabolic enzymes, and metabolomics of earthworms *Eisenia fetida*. Ecotoxicol. Environ. Saf..

[10] Ju Y.R., Su C.R., Chen C.F., Shih C.F., Gu L.S. (2024). Single and mixture toxicity of benzophenone-3 and its metabolites on *Daphnia magna*. Chemosphere.

[11] Rial D., Vazquez J.A., Menduina A., Garcia A.M., Gonzalez M.P., Miron J., Murado M.A. (2013). Toxicity of binary mixtures of oil fractions to sea urchin embryos. J. Hazard. Mater..

[12] Gaur J.P., Singh A.K. (1990). Effects of Petroleum Oils and Their Paraffinic, Asphaltic, and Aromatic Fractions on Photosynthesis and Respiration of Microalgae. Ecotoxicol. Environ. Saf..

[13] Li F., Yu Y., Guo M., Lin Y., Jiang Y., Qu M., Sun X., Li Z., Zhai Y., Tan Z. (2021). Integrated analysis of physiological, transcriptomics and metabolomics provides insights into detoxication disruption of PFOA exposure in *Mytilus edulis*. Ecotoxicol. Environ. Saf..

[14] Shi Z., Wen M., Zhang J., Tang Z., Wang C. (2020). Effect of phenanthrene on the biological characteristics of earthworm casts and their relationships with digestive and anti-oxidative systems. Ecotoxicol. Environ. Saf..

[15] Chen L., Bai J., Wan J., Song Y., Xiang G., Duan R., Zheng Y. (2024). Endocrine system, cell growth and death, and energy metabolism induced by Sb(III) exposure in earthworm (*Pheretima guillemi*) revealed by transcriptome and metabolome analysis. Environ. Pollut..

[16] Ji Q., Han L., Zhang T., Xia X., Xiang X. (2023). α-Linolenic acid alleviates aluminum toxicity in RAW264.7 cells by antioxidative and anti-inflammatory effects. Arab. J. Chem..

[17] Sun N., Liu Q., Wang J., He F., Jing M., Chu S., Zong W., Liu R., Gao C. (2021). Probing the biological toxicity of pyrene to the earthworm *Eisenia fetida* and the toxicity pathways of oxidative damage: A systematic study at the animal and molecular levels. Environ. Pollut..

[18] Reyes J.S., Cortes-Rios J., Fuentes-Lemus E., Rodriguez-Fernandez M., Davies M.J., Lopez-Alarcon C. (2024). Competitive oxidation of key pentose phosphate pathway enzymes modulates the fate of intermediates and NAPDH production. Free Radic. Biol. Med..

[19] Fuentes-Lemus E., Reyes J.S., Figueroa J.D., Davies M.J., López-Alarcón C. (2023). The enzymes of the oxidative phase of the pentose phosphate pathway as targets of reactive species: Consequences for NADPH production. Biochem. Soc. Trans..

[20] Kierans S.J., Taylor C.T. (2024). Glycolysis: A multifaceted metabolic pathway and signaling hub. Am. Soc. Biochem. Mol. Biol..

[21] Li X., Xiong D., Ju Z., Xiong Y., Ding G., Liao G. (2021). Phenotypic and transcriptomic consequences in *zebrafish* early-life stages following exposure to crude oil and chemical dispersant at sublethal concentrations. Sci. Total Environ..

[22] Wu Z.Z., Gan Z.W., Zhang Y.X., Chen S.B., Gan C.D., Yang K., Yang J.Y. (2023). Transcriptomic and metabolomic perspectives for the growth of alfalfa (*Medicago sativa* L.) seedlings with the effect of vanadium exposure. Chemosphere.

[23] Han Y., Ling S., Hu S., Shen G., Zhang H., Zhang W. (2024). Combined exposure to decabromodiphenyl ether and nano zero-valent iron aggravated oxidative stress and interfered with metabolism in earthworms. Sci. Total Environ..

[24] Brown S.A.E., Simpson A.J., Simpson M.J. (2009). 1H NMR metabolomics of earthworm responses to sub-lethal PAH exposure. Environ. Chem..

[25] Li Y., Zhang J., Zhang Y., Zhang B., Wang Z., Wu C., Zhou Z., Chang X. (2023). Integrated metabolomic and transcriptomic analysis reveals perturbed glycerophospholipid metabolism in mouse neural stem cells exposed to cadmium. Ecotoxicol. Environ. Saf..

[26] Wu C., Fang F., Yu Y., Wang B., Gao H., Cui W. (2023). Multi-omics analyses of serum metabolome, urine metabolome and gut microbiome reveal dysregulated glycerophospholipid metabolism in subacute cadmium-exposed wistar rats. Toxicology.

[27] Lu J., Quan J., Zhou J., Liu Z., Ding J., Shang T., Zhao G., Li L., Zhao Y., Li X. (2024). Combined transcriptomics and metabolomics to reveal the effects of copper exposure on the liver of rainbow trout (*Oncorhynchus mykiss*). Ecotoxicol. Environ. Saf..

[28] Yan L., Li S., Riaz M., Jiang C. (2021). Proline metabolism and biosynthesis behave differently in response to boron-deficiency and toxicity in *Brassica napus*. Plant Physiol. Biochem..

[29] Sun R., Xu K., Ji S., Pu Y., Man Z., Ji J., Chen M., Yin L., Zhang J., Pu Y. (2020). Benzene exposure induces gut microbiota dysbiosis and metabolic disorder in mice. Sci. Total Environ..

[30] Everitt S., Fujita K.K., MacPherson S., Brinkmann M., Pyle G.G., Wiseman S. (2021). Toxicity of Weathered Sediment-Bound Dilbit to Early Life Stages of Zebrafish (*Danio rerio*). Environ. Sci. Technol..

[31] Fujita K.K., Xia Z., Tomy G., Montina T., Wiseman S. (2021). 1H NMR based metabolomic profiling of early life stage zebrafish (*Danio rerio*) exposed to a water-soluble fraction of weathered sediment-bound diluted bitumen. Aquat. Toxicol..

[32] Jones O.A.H., Spurgeon D.J., Svendsen C., Griffin J.L. (2008). A metabolomics based approach to assessing the toxicity of the polyaromatic hydrocarbon pyrene to the earthworm *Lumbricus rubellus*. Chemosphere.

[33] Zhu Y., Ma X., Su G., Yu L., Letcher R.J., Hou J., Yu H., Giesy J.P., Liu C. (2015). Environmentally Relevant Concentrations of the Flame Retardant Tris(1,3-dichloro-2-propyl) Phosphate Inhibit Growth of Female Zebrafish and Decrease Fecundity. Environ. Sci. Technol..

[34] Bart S., Pelosi C., Nélieu S., Lamy I., Péry A.R.R. (2019). An energy-based model to analyze growth data of earthworms exposed to two fungicides. Environ. Sci. Pollut. Res..

[35] Wang P., Ng Q.X., Zhang B., Wei Z., Hassan M., He Y., Ong C.N. (2019). Employing multi-omics to elucidate the hormetic response against oxidative stress exerted by nC60 on *Daphnia pulex*. Environ. Pollut..

[36] Zhang Y., Huang C., Zhao J., Hu L., Yang L., Zhang Y., Sang W. (2024). Insights into tolerance mechanisms of earthworms (*Eisenia fetida*) in copper-contaminated soils by integrating multi-omics analyses. Environ. Res..

[37] Wu Y., Xia Y., Hu A., Xiong G., Wu W., Shi L., Chen L., Guo X., Qiao Y., Liu C. (2024). Difference in muscle metabolism caused by metabolism disorder of rainbow trout liver exposed to ammonia stress. Sci. Total Environ..

[38] Kuo A., Hla T. (2024). Regulation of cellular and systemic sphingolipid homeostasis. Nat. Rev. Mol. Cell Biol..

[39] Li F., Xiang B., Jin Y., Li C., Ren S., Wu Y., Li J., Luo Q. (2020). Hepatotoxic effects of inhalation exposure to polycyclic aromatic hydrocarbons on lipid metabolism of C57BL/6 mice. Environ. Int..

[40] Boukir A., Aries E., Guiliano M., Asia L., Doumenq P., Mille G. (2001). Subfractionation, characterization and photooxidation of crude oil resins. Chemosphere.

[41] Alsaadi F., Hodson P.V., Langlois V.S. (2017). An Embryonic Field of Study: The Aquatic Fate and Toxicity of Diluted Bitumen. Bull. Environ. Contam. Toxicol..

[42] Pinheiro I.F., Bizarre L., Perles C.E., Feitosa F.X., de Sant’Ana H.B., de Tarso Vieira Rosa P., van der Geest C., Guersoni V.C.B. (2024). Exploring asphaltene aggregation: Model systems based on toluene-heptane mixtures. Fuel.

