# Supplementary material for: Metabolomic-Based Assessment of Earthworm (Eisenia fetida) Exposure to Different Petroleum Fractions in Soils
Source: Metabolites. 2025 Feb 5;15(2):97. doi: 10.3390/metabo15020097 (PMC11857538; doi:10.3390/metabo15020097)
Supplement: Supplementary file 1 [file metabolites-15-00097-s001.zip › metabolites-3406505-supplementary.pdf]

Supplementary Material

ST1 Four Representative Components of Petroleum

Selection of representatives: For the saturated hydrocarbon components, both short-chain and long-chain n-alkanes, as well as cycloalkanes, were selected. For the aromatic hydrocarbon components, aromatic compounds with varying numbers of benzene rings were selected. The mixed components were prepared in equimolar ratios. Resin and asphaltene components were isolated from petroleum samples sourced from the Shengli Oilfield through column chromatography.

Saturated Hydrocarbon Components: n-decane, cyclopentane, cyclohexane, n-icosane, n-tetracosane, and n-hexatriacontane.

Aromatic Hydrocarbon Components: benzene, toluene, naphthalene, phenanthrene, pyrene, and chrysene.

ST2 Measurement of SOD and MDA in Earthworms

Following a 7-day toxicity test, two surviving earthworms from three parallel samples of each treatment group were combined, rapidly frozen in liquid nitrogen to preserve the worms in a consistent state at the conclusion of the experiment. The mixed earthworm samples were freeze-dried and ground into a uniform powder.

SOD Measurement: SOD activity was assessed using the Beiyuntian Reagent Kit S0101S. A suitable amount of homogenized earthworm powder was weighed and added to the SOD sample preparation solution, following the instructions provided wirth the kit. Due to the coloration of the earthworm samples, a blank control with three replicates was included, and the calculations were based on the determined protein content.

MDA Measurement: MDA levels were quantified using the Beiyuntian Reagent Kit (S0131S). An appropriate amount of homogenized earthworm powder was weighed, and a 1:10 sample-to-buffer ratio was established using 50 mmol PBA buffer, followed by homogenization. The subsequent experimental steps were carried out according to the instructions provided in the kit, and calculations were based on the determined protein content.

BCA Protein Measurement: Protein content was measured using the Beiyuntian Reagent Kit (P0010S).

ST3 Extraction of Metabolites and LC-MS/MS Conditions

A precise amount of 50 ± 5 mg of the sample was weighed into a 2-mL centrifuge tube, and 400 μL extraction solvent (methanol:water = 4:1 (v:v)), containing four internal standards (L-2-chlorophenylalanine (0.02 mg/mL), etc.) was added to the tube. The samples were ground using a cryogenic tissue grinder for 6 min (−10°C, 50 Hz) and extracted by low-temperature ultrasound for 30 min (5°C, 40 kHz). The processed samples were allowed to sit at −20°C for 30 min and centrifuged for 15 min (13,000 *g*, 4°C). The supernatant was transferred into a sampling vial with an insert for analysis. Additionally, 20 μL of the supernatant from each sample was combined and used as a quality control sample. The LC-MS analysis was performed using an AB SCIEX ultra-high-performance liquid chromatography tandem time-of-flight mass spectrometry (UHPLC-Triple TOF) system.

Chromatographic Conditions: The chromatographic column used was an ACQUITY UPLC HSS T3 (100 mm × 2.1 mm i.d., 1.8 μm; Waters, Milford, USA). Mobile phase A

consisted of 95% water and 5% acetonitrile (with 0.1% formic acid). Mobile phase B consisted of 47.5% acetonitrile, 47.5% isopropanol, and 5% water (with 0.1% formic acid). The flow rate was 0.40 mL/min, injection volume was 10 μL, and column temperature was 45°C. Both positive and negative ion modes were employed for MS/MS detection of metabolites. Key parameters included a scanning range of 50–200 m/z, spray gas at 50 psi, auxiliary heating gas at 50 psi, curtain gas at 35 psi, ion source temperature at 500°C, and ionization voltages of +5500 V and −4500 V for positive and negative modes, respectively. A declustering potential of 80 V was applied. Dynamic exclusion was also used to eliminate unnecessary MS/MS data.

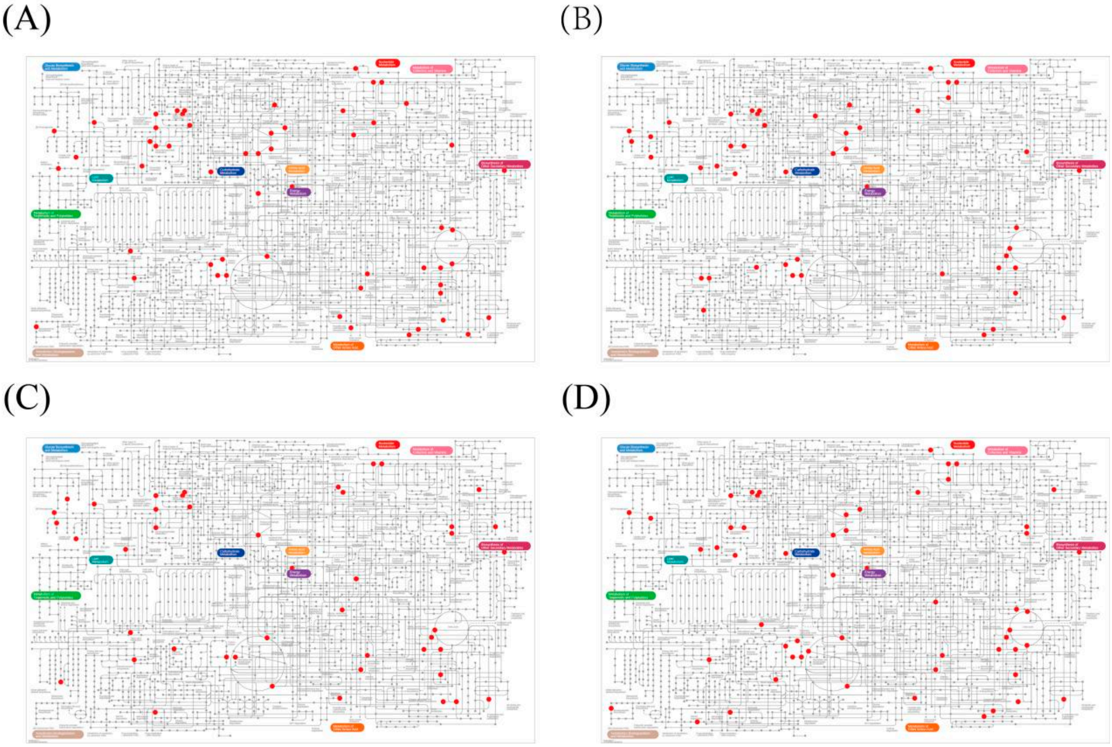

Figure S1. Diagram of iPath metabolic pathway analysis. Bolded dots in the graphs represent the variations in the metabolic pathway information for the whole biological system of earthworms by treatment groups. (A–D) represent saturated hydrocarbon, aromatic hydrocarbon, resin, and asphaltene fractions, respectively.

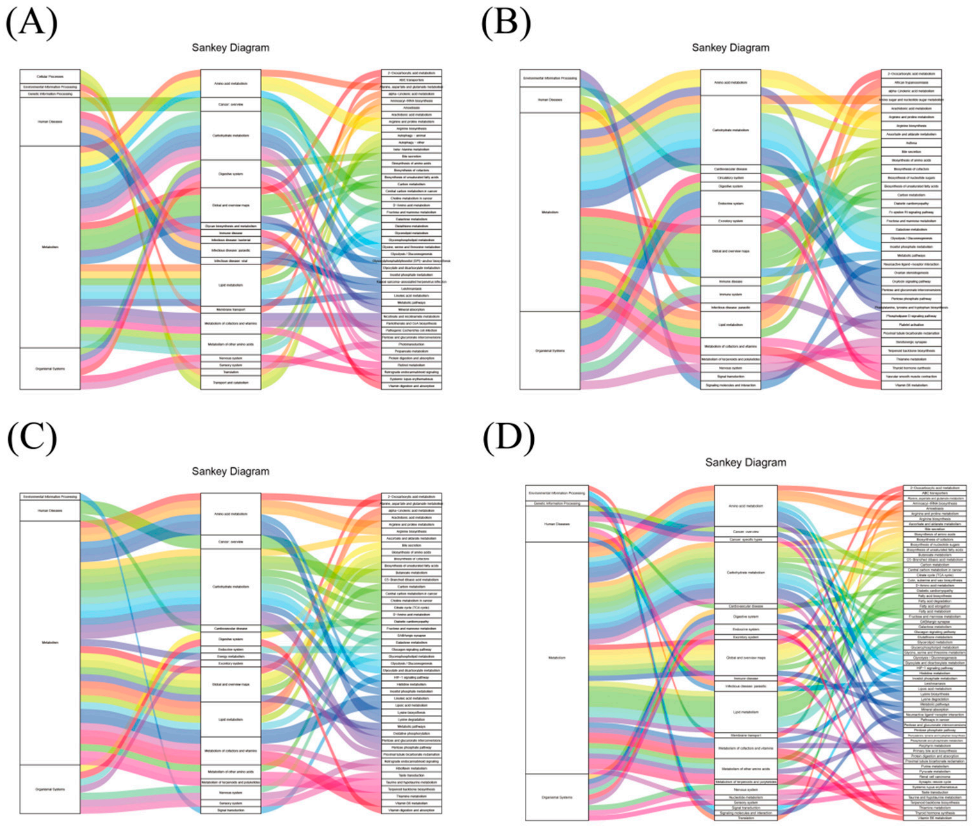

Figure S2. Sankey diagram of the first five differential metabolic pathways of the four components. (A–D) indicates the metabolic categories to which the five KEGG-enriched pathways belong before exposure to saturated hydrocarbon, aromatic hydrocarbon, gelatinous, and asphaltene fractions.

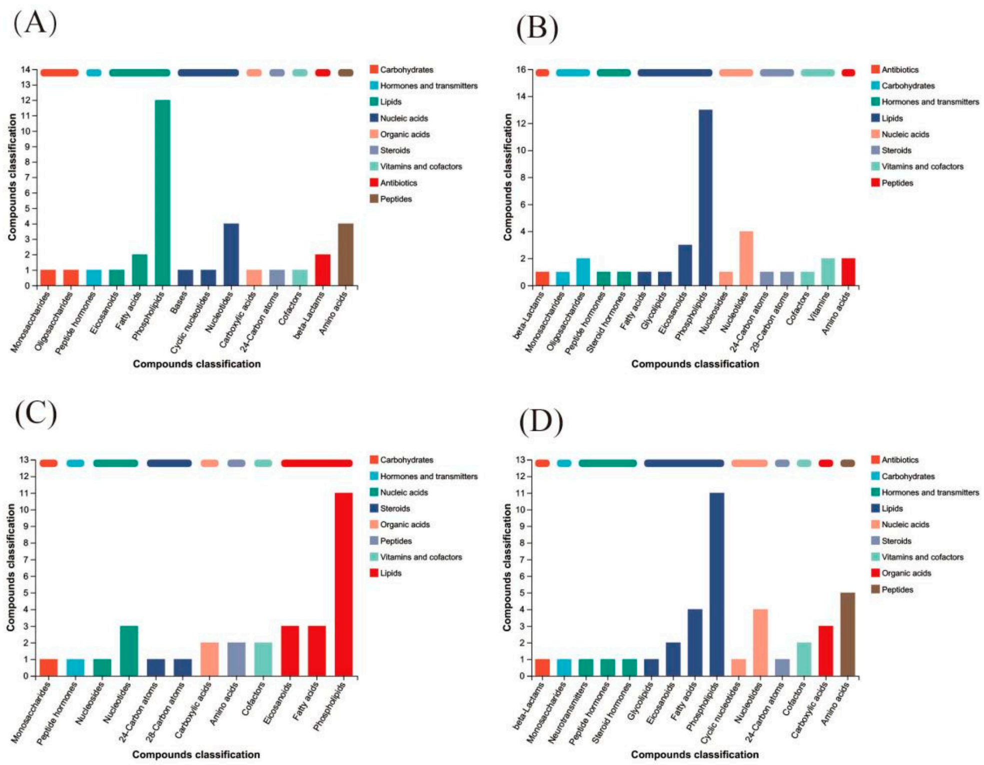

Figure S3. Individual difference plots for the four fractions. (A–D) indicate the significantly different metabolite categories between the four treatment groups and the control group.

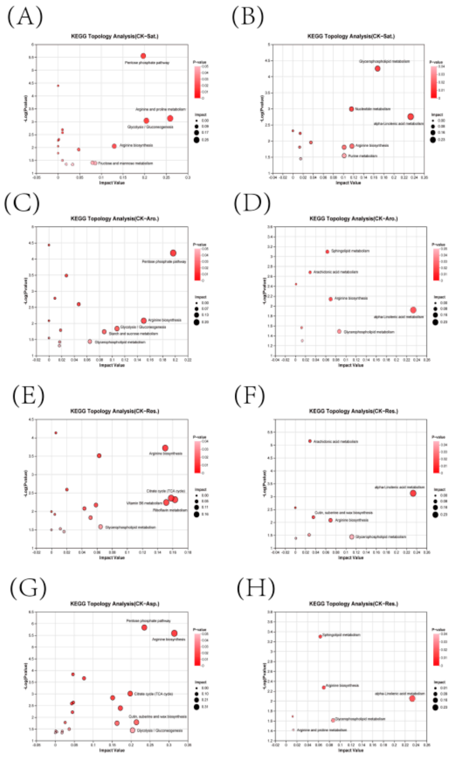

Figure S4. KEGG topology. (A–H) indicate significant pathways of upregulation and downregulation of different metabolites of saturated hydrocarbon, aromatic hydrocarbon, colloidal, and asphaltene fractions, compared with controls ($P < 0.05$)
